# Clinical efficacy of risankizumab in patients with psoriasis: A Japanese case series

**DOI:** 10.1016/j.jdin.2024.03.017

**Published:** 2024-04-06

**Authors:** Kanako Tsunoda, Daisuke Watabe, Hiroo Amano

**Affiliations:** Department of Dermatology, Iwate Medical University School of Medicine, Iwate, Japan

**Keywords:** biologics, efficacy, PASI score, psoriasis, real-world, risankizumab

*To the Editor:* To date, several reports have described the efficacy of risankizmab in actual clinical practice,^1^^,^^2^ yet there are no single-center study testing the efficacy in Asia. This study aimed to assess the efficacy of risankizumab on psoriasis in Asia. We conducted a case series study between June 2019 and May 2022, which was approved by the ethics committee of Iwate Medical University, Japan. We tested the differences in the proportions of the 2 groups using the χ^2^ test. Statistical significance was set at *P* < .01. Patient demographics are shown in Table I.

**Table I:** Dermographic and disease characteristics of the study population

Characteristics	*n* = 51
Age (y)	51.1 ± 12.9
Sex	
Male, *n* (%)	35 (68.6)
Female, *n* (%)	16 (31.4)
PsV, *n* (%): PsA, *n* (%)	44 (86.3): 7 (13.7)
BMI (kg/m^2^)	26.4 ± 4.7
BMI <25	20 (39.2)
BMI ≥25	31 (60.8)
Total psoriasis duration, y	
<1, *n* (%)	0
≥1-<5, *n* (%)	6
≥5-10<, *n* (%)	8
≥10-20, *n* (%)	16
≥20-30, *n* (%)	7
≥30, *n* (%)	5
Unknown/not recorded	9
Biologic-naïve, *n* (%)	28 (54.9)
Prior biologic use, *n* (%)	23 (45.1)
1 prior biologic, *n*	13
2 prior biologics, *n*	7
3 prior biologics, *n*	2
4 prior biologics, *n*	0
5 prior biologics, *n*	1
PASI at baseline	8.8 ± 6.0
DLQI score at baseline	6.8 ± 6.0

Data represent mean ± standard deviation for age BMI, PASI, and DLQI.

*BMI*, Body mass index; *DLQI*, Dermatology Life Quality Index; *PASI*, Psoriasis Area and Severity Index; *PsA*, psoriasis arthritis; *PsV*, psoriasis vulgaris.

We included 51 patients in our study, and none of them experienced any adverse events. We observed that the Psoriasis Area and Severity Index (PASI) score of 82.4% of the respondents improved by 75%, which accounted for PASI-75. Similarly, 47.1% and 29.4% of respondents showed improved PASI scores by 90% (PASI-90) and 100% (PASI-100) at week 16. At week 52, achievement rates were 81.1%, 75.7%, and 62.2%, respectively (Fig 1).

**Fig 1:**
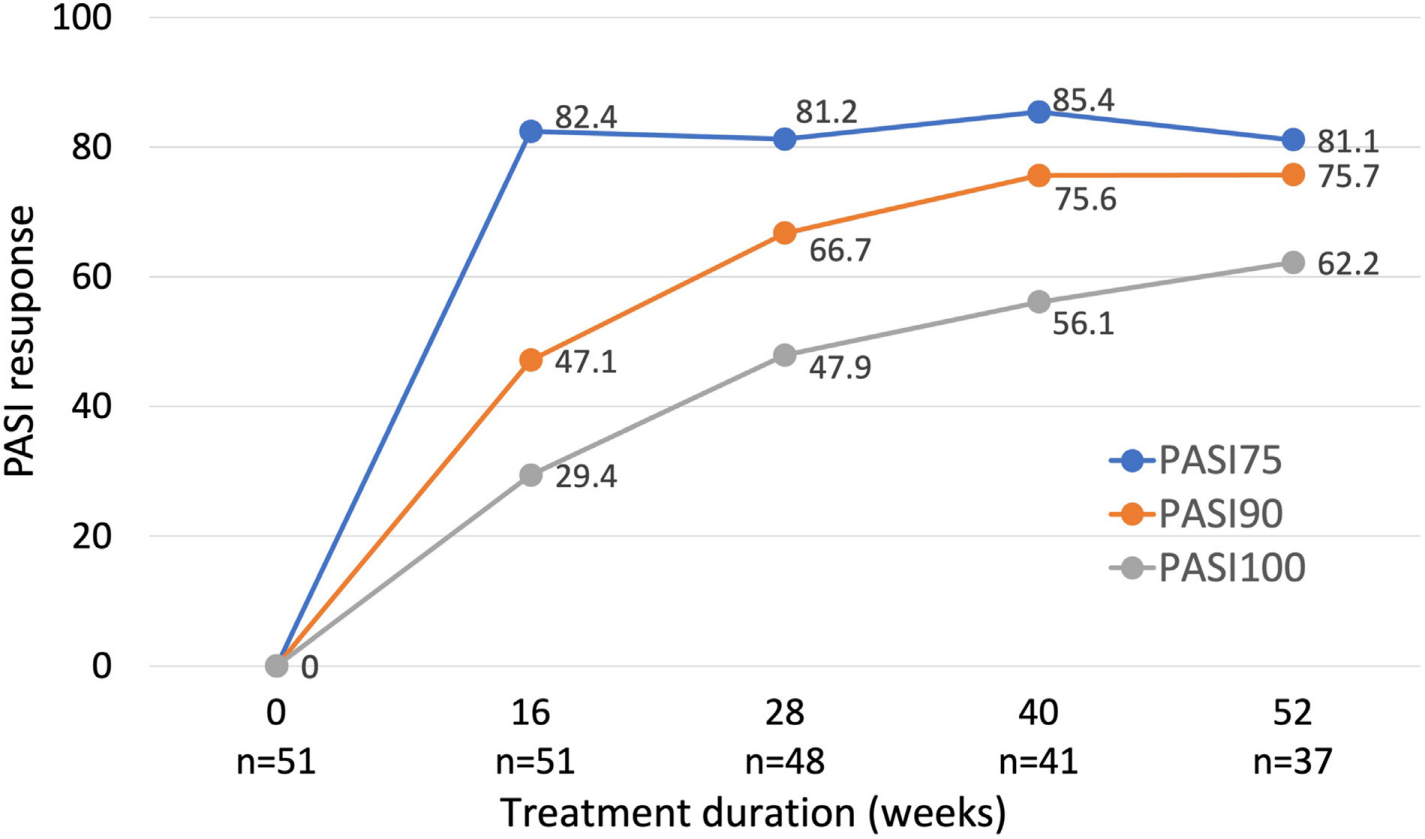
The following data indicates the percentage of patients who achieved PASI-75, PASI-90, and PASI-100 responses during the 52-week treatment. *PASI*, Psoriasis Area and Severity Index.

Moreover, at week 16, 94.1% and 54.9% of patients achieved absolute PASI scores of less than or equal to 3 and 1, respectively. At week 52, these percentages increased to 100% and 94.6%, respectively.

We also compared PASI-90 and PASI-100 at weeks 16 and 52 between bio-naïve and bio-experienced patients and between participants with a body mass index (BMI) less than 25 kg/m^2^ and BMI greater than or equal to 25 kg/m^2^. Bio-naïve patients tended to have higher achievement rates, and the difference from bio-experienced patients was only significant in the PASI-90 at week 52 (95.6% vs 57.6%, *P* < .01). Participants with a BMI less than 25 kg/m^2^ tended to have higher achievement rates. However, at week 52, the difference between the 2 groups decreased, particularly in the PASI-100.

Overall, all participants showed steady improvement over the study period. At week 40, all patients had a PASI score of less than or equal to 3, with the majority (90.2%) reaching a PASI score of less than 1.

In the UltlMMa-1 study,^3^ no significant difference in the PASI-90 achievement rate of risankizumab was observed in any of the subgroups, and the effect was reported to be unaffected by prior biologic therapy or high BMI. In our study, a significant difference was found only in the 52-week PASI-90 comparison, but basically bio-naïve tends to have a higher PASI improvement rate compared to bio-experienced. Also, a high BMI is a well-known negative predictor of psoriasis response to some biologic treatment.^4^^,^^5^ In our study, patients with a BMI of less than 25 tended to achieve higher PASI-90 and PASI-100 achievement rates at week 16, but at week 52, the difference between 2 groups narrowed. Although the sample size was small, and the study design was retrospective and observational, our current study provides real-world data on efficacy and safety of risankizumab.

## Conflicts of interest

None disclosed.

## References

[1] Gordon K.B., Strober B., Lebwohl M. (2018). Efficacy and safety of risankizumab in moderate-to-severe plaque psoriasis (UltIMMa-1 and UltIMMa-2): results from two double-blind, randomised, placebo-controlled and ustekinumab-controlled phase 3 trials. Lancet.

[2] Mastorino L., Susca S., Megna M. (2022). Risankizmab shows high efficacy and maintenance in improvement of response until week 52. Dermatol Ther.

[3] Borroni R.G., Malagoli P., Gargiulo L. (2021). Real-life effectiveness and safety of risankizumab in moderate-to-severe plaque psoriasis: a 40-week multicentric retrospective study. Acta Derm Venereol.

[4] Singh S., Facciorusso A., Singh A.G. (2018). Obesity and response to anti-tumor necrosis factor-α agents in patients with select immune-mediated inflammatory diseases: a systematic review and meta-analysis. PLoS One.

[5] Huang H., Cai M.L., Hong X.J. (2020). Real-world data on the use of sekukinumab as treatment for moderate-to-severe psoriasis in Chinese patients. Eur J Dermatol.

